# Screening Measures of Perinatal Mental Health and Wellbeing in Fathers: A Scoping Review

**DOI:** 10.3390/ijerph22071126

**Published:** 2025-07-16

**Authors:** Vincent Mancini, Yonatan Ambrosio Lomeli, Thomas P. Nevill, Thomas B. Marsh, Ezra Kneebone, Alka Kothari

**Affiliations:** 1 Human Development and Community Wellbeing, The Kids Research Institute Australia, Perth 6009, Australia; yonatana@alumni.princeton.edu (Y.A.L.); thom.nevill@thekids.org.au (T.P.N.); ezra.kneebone@thekids.org.au (E.K.); 2 Centre for Child Health Research, University of Western Australia, Perth 6009, Australia; 3 Discipline of Psychology, School of Population Health, Curtin University, Perth 6845, Australia; 4 Department of Psychology, Princeton University, Princeton, NJ 08540, USA; 5 Faculty of Health and Behavioural Sciences, University of Queensland, Brisbane 4072, Australia; alka.kothari@uq.edu.au; 6 Redcliffe Hospital, Metro North Health, Redcliffe 4020, Australia

**Keywords:** fathers, perinatal mental health, screening, instrument, assessment

## Abstract

Accurately screening fathers for perinatal mental health problems requires well-validated screening instruments that assess the expression of paternal perinatal mental distress. This study aimed to identify and describe the psychometric properties of perinatal mental health screening instruments administered to paternal cohorts within the past two decades. A scoping review was conducted following Arksey and O’Malley’s scoping review framework and is reported in line with the PRISMA-ScR guidelines. A systematic search of Embase, PsycINFO, Medline, and ProQuest databases identified peer-reviewed literature published within the past 20 years that implemented a screening instrument for fathers’ perinatal mental health within the first 12 months of their child’s birth. Twenty-eight instruments used to screen fathers’ perinatal mental health were identified across 36 studies. The instruments most frequently assessed symptoms of depression. Only five were explicitly developed for fathers and while these instruments produced promising results, further evaluation is necessary before they can be considered a superior screening method compared to existing instruments.

## 1. Introduction

Psychologists and mental health professionals working in perinatal mental health are routinely tasked with assessing and treating parents for mental health conditions that manifest during this often challenging period. As evidence continues to document the prevalence and consequences of perinatal mental health conditions amongst *fathers* (a term inclusive of any male primary caregiver), the need for timely and accurate identification of these conditions is critical [1,2,3]. Currently, there is no gold standard screening measure for perinatal mental health conditions validated for use in fathers. Accordingly, this study aims to identify and compare screening assessments validated for use in the paternal context. Before this, we provide a brief literature review on paternal perinatal mental health.

### 1.1. The Transition to Fatherhood

Becoming a parent gives rise to a newfound identity. For most men, this includes their new identification as a father [4,5]. This identity is not only defined by observable behaviors consistent with what it means to be a father (e.g., engaging in caregiving duties) but also by internal processes that see the development of new roles, responsibilities, and purpose in life [6,7]. This is particularly pertinent during the early transition into fatherhood (either for the first time or after another child’s birth) [4,7]. This period spans conception to ~12 months of their child’s life. This aligns with the United Kingdom’s National Health Service’s definition of the perinatal period published in 2014, which refers to the period from conception to one year after birth [8].

Fathers encounter numerous challenges during the perinatal period, such as adjusting to changes in their relationship with their partners, feeling unprepared for childbirth and parenting, inadvertent exposure to traumatic events, and experiencing exclusion from services [1,9,10,11,12]. These challenges contribute to men being at increased risk of poor mental health during the perinatal period.

### 1.2. Fathers’ Perinatal Mental Health

In this paper, we use the term ‘perinatal mental health’ to encompass mental health conditions, psychological distress, and psychological wellbeing during the perinatal period. This aligns with the World Health Organization’s understanding of mental health, published in 2014, and reflects the shift in mental health care from solely addressing mental health issues to promoting psychological wellbeing [13,14].

Contemporary understandings of perinatal mental health have largely been influenced by earlier research focused on the experiences of birthing mothers [15,16,17]. There is a comparatively small but growing amount of research specifically focused on fathers’ perinatal mental health. Like the maternal literature, much of the literature exploring men’s perinatal mental health has been examined through a diagnostic lens and has commonly focused on postnatal depression (PND) [18,19,20]. Current prevalence estimates suggest that as many as 1 in 10 men worldwide experience clinically significant symptoms of depression during this period—a condition commonly described by researchers as Paternal Perinatal Depression (PPND) [21]. However, many more may experience significant impairments below clinical cut-offs or that are characterized by symptoms that are not consistent with postnatal depression yet nevertheless impact their perinatal mental health [22]. Thus, the absence of a mental health condition (e.g., postnatal depression) does not guarantee the absence of poor perinatal mental health for new fathers [23]. Studies have also identified that men are less likely to report their experiences of poor mental health during the perinatal period, which may partly explain discrepancies in reported prevalence [24].

Beyond PPND, fathers may experience additional affective and behavioral concerns that may not always be clinically diagnosable. These concerns include symptoms of anxiety, anger and frustration, emotional dysregulation, substance abuse and risk-taking behavior, somatic symptoms, and even suicidality [11,25,26,27]. In a systematic review published in 2017, Philpott et al. reported that fathers experienced significant stress during the perinatal period, especially leading up to their child’s birth, which contributed to mental health issues such as anxiety, psychological distress, and fatigue [2].

The consequences of poor perinatal mental health are pervasive [23]. A 2020 systematic review noted the growing amount of research identifying increased developmental risk for infants with fathers experiencing perinatal mental health difficulties, especially depression [3]. In their 2017 review of research on the impact of untreated paternal depression during the perinatal period, Gentile and Fusco found that there were small but significant associations between paternal depression during pregnancy and/or after childbirth and children’s behavioral, emotional, and social functioning at 36 months, and the development of psychiatric disorders in children by seven years [28]. These associations were adjusted for maternal depression. Relatedly, a longitudinal study published in 2018 found that fathers experiencing postpartum depressive symptoms had a poorer attachment to their infants [29]. This diminished attachment was found to significantly impact the infants’ later social development, thereby mediating the association between paternal postpartum depression and adverse infant social development [29]. The impact of poor perinatal mental health on both fathers and their children emphasizes the importance of ensuring that there are appropriate mental health screening procedures in place for fathers during this period.

### 1.3. Screening Fathers’ Perinatal Mental Health

The mental health of fathers and non-birthing parents is not always included as part of routine perinatal care. However, advances in research, policy, and advocacy have contributed to gradual shifts toward the inclusion of mental health screening for all parents. For example, Australia’s Centre of Perinatal Excellence (COPE) provided screening and psychosocial assessment recommendations for fathers and non-birthing parents as part of their 2023 National Perinatal Mental Health Guidelines [30]. Such guidelines exemplify a shift toward the inclusion of managing fathers’ perinatal mental health as part of family-centered care.

Fundamental to effective interventions addressing fathers’ perinatal mental health is the ability to accurately identify vulnerable or at-risk individuals. It is, therefore, essential to have reliable and valid screening measures for paternal perinatal mental health. These measures can identify those at risk or vulnerable and help monitor the effectiveness of interventions. The current literature evaluating measures to screen and assess parental mental health during the perinatal period commonly focuses on the Edinburgh Postnatal Depression Scale (EPDS). The EPDS is one of the most widely used and empirically validated screening instruments for postnatal depression in mothers [31,32,33]. Modified cut-off scores are used for fathers to compensate for gendered differences in how men and women respond to certain scale items, such as those that refer to crying [34,35]. This reflects an acknowledgment that male and female expressions of depression may vary, though whether the retained symptoms in the EPDS comprehensively describe men’s experiences is not clear [36,37].

A recent systematic scoping review of instruments focused on paternal postpartum depression by Berg et al. analyzed 59 studies and found that out of 13 different measures of postpartum depression identified, the EPDS was used most frequently [38]. These authors also recognized that none of the identified measures were uniquely developed with paternal depressive symptoms in mind. However, the review focused explicitly on assessments of depression only rather than the broader constellation of psychological distress or impairment during this period.

Our evolving understanding of the paternal experience of perinatal mental health has generated curiosity about whether the modified scoring procedures of the EPDS offer sufficient content validity and accuracy in identifying fathers at risk of poor mental health in the perinatal period. This argument is supported by the broader psychopathology literature from the past two decades that has identified significant differences in the male and female expression of depression [39,40]. For example, the male expression of depression is more likely to include symptoms of impulsivity and alcohol and substance abuse—behavioral indicators that are not readily captured in the EPDS. Additionally, the use of any instrument measuring depressive symptoms in the perinatal period may not be able to offer insights into other forms of psychosocial distress or impairments that may exert similar consequences upon parents, children, and families [41].

One example of a screening measure designed to address these limitations is the Perinatal Assessment of Paternal Activity (PAPA) scale developed by Baldoni et al. [21]. This screening instrument aims to precisely capture male experiences of perinatal depression while also assessing a broader constellation of affective disorders to identify fathers who may need additional support. Whether such measures are superior, inferior, or simply different from the EPDS in assessing paternal perinatal mental health is a subject for future research. Further investigation may help to identify novel and nuanced ways to identify fathers at risk for poor perinatal mental health during the perinatal period—thus driving better opportunities for timely and targeted support. However, it is essential first to assess what screening assessments have been used to evaluate fathers’ perinatal mental health.

### 1.4. Prior Reviews

This paper expands on recent reviews by focusing on screening measures used to assess different aspects of men’s mental health during the perinatal period. We aim to go beyond recently published reviews by not only focusing on screening measures for identifying postpartum depression but also including measures used for other conditions [38,42]. This broader approach is essential because men experience other mental health issues during the perinatal period in addition to depression, and whether other well-validated measures assess this broader construction of perinatal mental health is not clear. Other reviews have focused on measures used to screen for parental stress (including fathers) in the postpartum period [43], while Brekke identified Quality of Life instruments for parents (including fathers) during pregnancy and the postpartum period [44]. Although these reviews provide valuable insights, they do not specifically focus on screening measures for fathers, nor do they concentrate exclusively on the perinatal period.

### 1.5. The Present Review

There is growing recognition amongst healthcare providers, scholars, and the community about the need to identify and support the perinatal mental health of fathers—such as that recognized in the Western Australian Perinatal and Infant Mental Health Model of Care published in 2016 [45]. The successful implementation of any policies to achieve this aim hinges on the ability to identify those at-risk or experiencing poor perinatal mental health. This could be achieved through the implementation of routine screening measures as part of routine pregnancy care. However, for this to be successful, the measure must be psychometrically sound, i.e., able to accurately measure the phenomenon of interest (validity) and do so consistently (reliability). Where reliability captures different dimensions of consistency (e.g., consistency in response to items across time and informants), validity is broadly concerned with how an instrument measures the underlying concept or condition it was designed to measure.

Thus, a review of the extant literature is a timely one. The scoping review addressed the following questions: “Which instruments are used to screen paternal mental health during the perinatal period?” and “What are their corresponding characteristics and psychometric properties?”

## 2. Materials and Methods

The scoping review was determined to be the most appropriate form of evidence synthesis to achieve the aims of the present study, given its proficiency in summarizing existing research, identifying research gaps, and informing directions for future research activities [46]. Compared to systematic reviews and meta-analyses, the scoping review adopts a broader approach better equipped to describe, for example, the types of measurement instruments used across research studies to capture a given phenomenon. Scoping reviews adhere to the same structured and transparent process as systematic reviews [47].

We followed the five-stage methodological framework to conduct high-quality scoping reviews as outlined by Arksey & O’Malley in 2005 and updated by Levac and colleagues in 2010 [46,48], described below.

### 2.1. Protocol Registration

The protocol for this scoping review was registered on the Open Science Framework (OSF) (Available online: https://doi.org/10.17605/OSF.IO/X39BT (accessed on 7 June 2025)) in November of 2023.

### 2.2. Eligibility Criteria

The population, concept, context, and type of evidence described below were used to determine article eligibility for this review.

#### 2.2.1. Population

The review included articles that focused on screening and assessment tools used in paternal populations. Accordingly, this scoping review included fathers or any other primary male figure (e.g., ‘stepfathers,’ ‘non-biological fathers,’ and ‘father figures’). Studies that did not emphasize screening specifically for fathers or used general parental scales without separating fathers from mothers were excluded.

#### 2.2.2. Concept

The scoping review focused on screening and measurement tools (e.g., scales, questionnaires, etc.). Therefore, only studies that analyzed the measurement of paternal perinatal mental health were selected.

#### 2.2.3. Context

This review did not apply clinical, cultural, or geographic limits. We limited eligible studies to the perinatal period for the father (the pregnancy of the partner up until 12 months after the child’s birth).

#### 2.2.4. Sources of Evidence

Peer-reviewed studies published between January 2003 and June 2023 were eligible for inclusion. Only peer-reviewed journal articles were included to ensure the review’s feasibility, limit the breadth of sources, and ensure that any records were subject to the peer-review process. The review did not include alternative forms of literature (e.g., book chapters, news articles, editorials, protocols, opinion papers, and conference proceedings) but these were used to identify other potential studies. Studies published from 2003 onwards were included to provide the most recent and up-to-date review of the existing literature.

### 2.3. Identifying Relevant Studies

A systematic search spanning Embase, PsycINFO, Medline, and ProQuest was conducted in July 2023. The selection of these databases was justified by prior evidence published in 2016 and 2017 supporting the comprehensiveness of these databases for systematic reviews in mental health and related disciplines, with particular support for Medline for use in reviews on mental health screening instruments [49,50]. The search strategy was developed using combinations of Boolean phrases and truncation strategies to expand and narrow the search for relevant publications.

We used generative predictive text (GPT-3) software to refine the search terms further. Specifically, GPT-3 was given the following prompt, “*I am conducting a scoping review. The review will be about assessment tools used to measure perinatal mental health and adjustment used in samples of men, males, fathers, and dads. Generate search terms for Embase, Medline, ProQuest, and PsycINFO*”. The responses to these prompts were used to refine the final search terms used for each database and identify any additional terms or Boolean operators not included initially (see Supplementary Materials).

Before collecting the full results from each database, a pilot search was performed to verify whether key studies (*n* = 10) known to the researchers via previous familiarization of the literature could be identified using the search terms developed. Pilot testing revealed that these ten articles were identified across the four databases, supporting the quality of the study search terms. The complete list of search terms was as follows:

((“assessment tool” OR “measurement instrument” OR “questionnaire” OR “scale” OR “inventory” OR “instrument” OR “psychometric” OR “measurement” OR “psychological test”) AND (“perinatal” OR “postpartum” OR “antenatal” OR “prepartum” OR “antepartum” OR “postnatal” OR “pregnancy” OR “birth” OR “childbirth”) AND (“mental health” OR “wellbeing” OR “adjustment” OR “depression” OR “depressive” OR “anxiety” OR “anxious” OR “psychopathology”) AND (“men” OR “males” OR “fathers” OR “dads”)) AND (TI: men OR TI: males OR TI: fathers OR TI: dads OR AB: men OR AB: males OR AB: fathers OR AB: dads).

### 2.4. Selecting Relevant Studies

Results were exported from the databases into Rayyan QCRI and deduplicated [51]. Two reviewers used Rayyan to independently screen each article’s titles and abstracts. Blind mode was used, where both reviewers could screen the same dataset at the same time without overlap. Afterwards, any disagreements on whether a study should be included in the review were resolved via re-examination of the title and abstract and a discussion between the two reviewers with an additional reviewer to resolve any outstanding conflicts. The same two reviewers then independently reviewed the full-texts of potentially eligible studies, with any disagreements resolved as before.

### 2.5. Data Charting

A data charting table was developed to document the relevant details to extract from each included study. The table mirrors the Joanna Briggs Institute (JBI) template for data extraction and was adapted for the current study context. The table included citation details, country, context, participant details, the instrument used, mental health domains assessed, number of items in the instrument, details of any psychometric validation, and who completed the screening instrument (e.g., self-report, partner report, clinician report).

### 2.6. Collating, Summarizing, and Reporting Findings

Instruments used in the studies were divided into two categories: (i) instruments specifically developed to measure perinatal mental health and (ii) instruments not specific to the perinatal period but measuring broader mental health and wellbeing administered during the perinatal period. Different versions of the same instruments (e.g., instruments that were revised or updated) were regarded as the same instrument. Qualitative descriptions of the instruments and their psychometric properties (instrument validity and reliability) were produced to complement the quantitative description of the pattern of findings (e.g., the frequency of instruments used across studies).

## 3. Results

### 3.1. Literature Search

**Figure 1:**
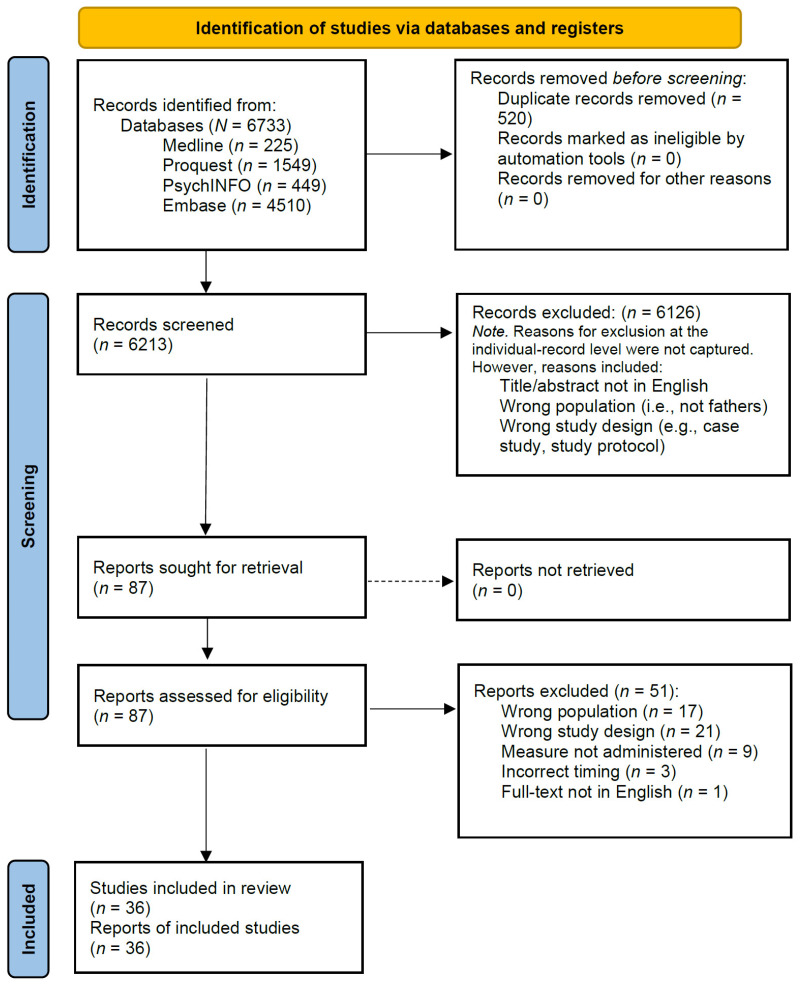
A 2020 PRISMA Flow Diagram outlining the study selection process [52].

The search initially produced 6733 articles from the four chosen databases (Figure 1). Two reviewers independently reviewed all texts to determine eligibility. Overall ratings of agreement between the two independent reviewers were high, with initial consensus (i.e., include’ or ‘exclude’ for full-text screening exceeding 90% of all texts). Following de-duplication, 6213 articles were identified to undergo title and abstract screening, yielding 87 articles that met the inclusion criteria for full-text screening. Records excluded at the title and abstract phase included those that fell clearly outside of the study inclusion criteria. Fifty-one studies did not meet the inclusion criteria following full-text screening and were rejected for reasons described in Figure 1. In total, 36 unique studies met the inclusion criteria and were retained for this review (Table 1).

### 3.2. Characteristics of the Included Studies

The included studies were conducted across 15 countries (Table 2). Most studies were conducted in Sweden (*n* = 6), followed by Australia, Italy, the United Kingdom (*n* = 4 each), and the United States (*n* = 3). Other studies were conducted in China, Iran (*n* = 2 each), Canada, Chile, Denmark, Germany, Malaysia, Portugal, Taiwan, and Vietnam (*n* = 1 each). Some studies were conducted in multiple countries (*n* = 3). All studies included at least one self-report assessment of fathers’ mental health during the perinatal period—with one study also including a partner report. One third of studies measured perinatal mental health during pregnancy (*n* = 9), while the remaining two thirds measured postpartum. Of these latter studies, half measured at 0–6 months (*n* = 9) and half at 6–12 months postpartum (*n* = 9).

### 3.3. Characteristics of the Instruments

Across these 36 studies, 28 different instruments were used to measure fathers’ mental health dimensions across the perinatal period. Fourteen of these instruments were specifically developed to measure perinatal mental health while the remaining (*n* = 14) were broader assessments of mental health that were validated or administrated during the perinatal period.

#### 3.3.1. Perinatal Mental Health Instruments: Characteristics

There were 14 instruments with data reporting their ability to measure perinatal mental health specifically, evidenced by the inclusion of items that are directly relevant to the pregnancy-to-perinatal period (e.g., the Fathers Postnatal Health Instrument Questionnaire) or with clear and theoretically driven rationale for items that did not make specific reference to children but pertained to the experience of perinatal mental health difficulties (e.g., the EPDS). As anticipated, over half (20 out of 36) of the included studies used the EPDS. The only other measure specific to the perinatal period used across multiple studies was the Father’s Fear of Childbirth Scale (*n* = 2). The 12 remaining measures were uniquely assessed in one study each. These were the Birth Experiences Questionnaire, Fathers Postnatal Health Instrument Questionnaire, First Time Fathers Experience of Childbirth, Karitane Parenting Confidence Scale, Parents Postnatal Sense of Security Instrument, Paternal Adjustment and Paternal Attitudes Questionnaire, Perinatal Assessment of Paternal Affectivity, Pregnancy-Related Anxiety Scale, Psychosocial Questionnaire, Salmon’s Item List, The Blues Questionnaire, and the City Birth Trauma Scale.

These screening instruments ranged from 8 to 30 items. Perinatal mental health was assessed via self-report for all 14 instruments. However, one study evaluated paternal symptoms of postnatal depression using the EPDS obtained data via partner report [77]. Each of these instruments and the supporting studies describing them are reported in Table 3. Five of these instruments, Fathers Postnatal Health Instrument Questionnaire, Father’s Fear of Childbirth Scale, First Time Fathers Experience of Childbirth, Paternal Adjustment and Paternal Attitudes Questionnaire, and Perinatal Assessment of Paternal Affectivity, were designed for specific administration amongst the paternal population, whilst the remaining scales were originally conceptualized and validated for use in maternal samples.

#### 3.3.2. General Mental Health Instruments: Characteristics

Several studies that assessed the mental health of fathers during the perinatal period using screening instruments that are broadly suitable for the general population and non-specific to the parenting experience were also identified.

The literature search identified 14 unique instruments that reported data on the mental health of fathers between pregnancy and the first 12 months of their child’s development. The most frequently used screening instrument was the Beck Depression Inventory (*n* = 6), followed by the Center for Epidemiologic Studies Depression Scale (*n* = 3) and the Gotland Male Depression Scale (*n* = 3). The remaining 11 instruments were each described in only one published study. These were the General Health Questionnaire, Hospital Anxiety and Depression Scale, Impact of Event Scale-Revised, Kessler Psychological Distress Scale, Matthey Generic Mood Questionnaire, Patient Health Questionnaire: Depression Module, Perceived Stress Scale, Post Traumatic Stress Disorder Checklist for DSM-V, The Symptom Checklist 90-Revised, Visual Analogue Scales, Zung’s Self-Rated Anxiety Scale.

These screening instruments ranged from 4 to 90 items, with all available data derived exclusively from fathers’ self-reports. Table 4 presents details regarding the mental health domains assessed by each instrument and further details (e.g., duration, example items, and response format). None of these instruments were explicitly developed for use in fathers or parents more broadly. However, one instrument—the Gotland Male Depression Scale—was developed and validated for use in a male population.

### 3.4. Psychometric Properties of the Instruments

There were mixed levels of detail about the 28 instruments’ psychometric properties across the 36 included studies. Details on these psychometric properties are described separately for the instruments that specifically assessed paternal perinatal mental health and those that measured paternal mental health during the perinatal period—but not specific to the perinatal period.

#### 3.4.1. Perinatal Mental Health Instruments: Psychometric Properties

Twelve of the fourteen instruments that specifically assessed paternal perinatal mental health reported a measure of internal reliability in either Cronbach’s alpha or McDonald’s omega (see Table 5). Only the Psychosocial Questionnaire and Blues Questionnaire described in Fletcher et al. and Edhborg did not indicate internal reliability [83,84]. Among the studies that did indicate internal reliability, the reported coefficients often demonstrated acceptable levels (α ≥ 0.70). Only two of the fourteen instruments identified in this section reported on the temporal stability of the instrument over time. These were the Father’s Postnatal Health Instrument Questionnaire over a one-week interval and the Perinatal Assessment of Paternal Affectivity over three months [21,78]. Both instruments reported good test re-test reliability across their prospective time points. Inter-rater reliability was noticeably absent for each instrument, which was perhaps unsurprising given the reliance on self-reported data. The construct validity of 12 of the 14 instruments was investigated via some form of factor analytic approach (e.g., confirmatory factor analysis, exploratory factor analysis)—the Psychosocial Questionnaire and Blues Questionnaire described in Fletcher et al. and Edhborg [83,84], respectively, did not include an indication of construct validity or factor structure. Ten of the fourteen instruments also indicated instrument criterion validity in the form of concurrent or predictive validity (see Table 5). Specifically, concurrent validity was frequently assessed by studies that report on multiple assessments of perinatal mental health and reporting bivariate correlations between obtained scores, with available data for these 12 instruments supporting the criterion validity of these instruments (see Table 5).

#### 3.4.2. General Mental Health Instruments: Psychometric Properties

Details on the psychometric data reported for each instrument are in Table 6. Congruent with the previous instruments, internal reliability was the most frequently reported metric, included for 11 of the 14 instruments. A measure of internal reliability was not reported in the studies that evaluated the Kessler Psychological Distress Scale [64], Matthey Generic Mood Questionnaire [63], and Visual Analogue Scales in their cohorts of fathers screened during the perinatal period [83]. Measures of test, re-test, and inter-rater reliability were noticeably absent for all 14 instruments. Four instruments reported limited construct validity evidence by calculating receiver operating characteristics [76,80]. One study by Psouni et al. [71], which included the Gotland Male Depression Scale and the EPDS, used exploratory factor analysis to develop a composite scale constituting items in both scales. Eight instruments reported details of criterion validity, most frequently via reported correlations with the EPDS (*n* = 6 of the eight instruments evaluated tested criterion validity against the EPDS). Overall, there is limited information on the reliability and validity of these instruments for use with fathers in the perinatal period.

## 4. Discussion

This review identified 28 instruments across 36 peer-reviewed and published studies that screened fathers’ mental health within the paternal population during the perinatal period. Fourteen of these instruments were developed specifically for use amongst new and expecting parents. The remaining fourteen screening instruments administered to fathers during the perinatal period were not initially designed for specific use in the perinatal context. Instead, these were broader assessments of mental health (e.g., depression) that were administered to a perinatal population. Few of the included studies described development or validation procedures for the experiences of fathers (or non-birthing parents, more broadly) in the perinatal period. Therefore, the majority of included studies were primarily underpinned by certain assumptions, such as that (a) fathers’ perinatal mental health can be reliably assessed using the same (or similar) procedures that are used to assess mothers’ perinatal mental health, and/or (b) the assessment of perinatal mental health difficulties can be achieved using measures that were not specifically developed to capture the unique experiences of this period. The results help assess the current literature and whether these assumptions are valid.

### 4.1. Decoupling Perinatal Depression from Poor Perinatal Mental Health

Of the included studies, most focused on the expression of symptoms consistent with the expression of depression. This result is somewhat expected, given the historical emphasis that has been placed on maternal depression—and, more recently, anxiety—during the postnatal and perinatal periods.

Throughout the review process, one interesting observation was a tendency for articles to use terminology such as “mental health” in the postnatal and perinatal periods to be synonymous with depressive disorders, either in their titles, abstracts, or text. This observation is not limited to those studies included in this review, with several recent examples of published literature on paternal ‘perinatal mental health’ concentrating, often exclusively, on the experience of depression [1,22,109]. One unintended consequence of this preoccupation with the onset of perinatal depression is that other types of poor mental health may remain unidentified. Importantly, the absence of depression in the perinatal period should not be mistaken for the lack of poor mental health. One practical recommendation for the field is that scholars and clinicians attempt to specify the dimensions of mental health under investigation.

It is essential to acknowledge recent efforts to advance the identification of perinatal psychological disorders in fathers. For example, the work of Baldoni and Giannotti provided a compelling argument for the need to “rethink perinatal psychological disorders considering the wide array of paternal affective symptoms and the limitations of current tools developed to assess maternal depression” [25] (p. 2). Their research led to the development of a new screening instrument tailored toward the male experience of a broader constellation of affective disorders (in addition to depression) during the perinatal period, the Perinatal Assessment of Paternal Affectivity (PAPA) [21]. PAPA was originally developed for the Italian population and has recently been validated in a Chilean population [110]. The development of innovative and contemporary screening instruments is welcomed. However, they remain subject to further evaluation before being considered a superior screening method compared to existing instruments. For example, researchers may first need to identify whether an instrument such as the PAPA provides a more accurate identification of at-risk fathers than other established instruments (e.g., the EPDS). Moreover, it will also be essential to determine whether clinicians and healthcare professionals deem such instruments appropriate and viable for use in the field, as these beliefs govern screening practices [111].

### 4.2. Are Common Screening Instruments Capturing Common Symptoms of Poor Mental Health in Fathers?

Prior research has suggested that the behavioral manifestation of depression amongst males is more frequently expressed via symptoms of irritability, impulsivity, or alcohol/substance abuse [39,40]. These behaviors are notably absent from the EPDS, which was the most used assessment of paternal mental health identified in this study and other reviews specific to paternal perinatal depression [38,112]. Whether items in the EPDS capture the full range of behaviors commonly expressed by males—and thus a valid instrument for use in this group—is unclear [71]. Assessing a smaller number of depressive symptoms could inadvertently lead to the underreporting of significant impairments that might instead manifest as other symptoms not measured by the instrument but potentially just as impairing. Though the EPDS is an illustrative example, it should not be interpreted as a criticism of this instrument. Instead, we hope these findings and discussion will invite further innovation in assessing perinatal mental health, creating opportunities to identify and support the community. Consensus regarding how we should be screening for perinatal mental health difficulties among fathers may be an important early step to inform the development and delivery of appropriate screening measures.

### 4.3. The Reliability and Validity of Mental Health Screening Instruments Require Further Development When Administered to Fathers

The psychometric properties of instruments purposefully designed to capture perinatal mental health (e.g., EPDS) were more frequently reported (Table 5) compared to instruments that were not specifically tailored to the paternal perinatal experience but administered to this cohort, such as the BDI-II (Table 6). This is likely because most instruments capturing perinatal mental health in fathers were validation studies, where reporting these additional metrics is standard practice [21]. Another compelling reason for the general lack of psychometric evaluation amongst the non-perinatal-specific assessments of mental health is due to the well-established properties of these measures outside of the paternal and perinatal contexts. Although not specific to the paternal perinatal population, these large bodies of evidence may inadvertently drive the assumption that the instrument will operate similarly in this cohort. Instruments such as the BDI-II have been extensively validated across populations, cultures, and contexts [113,114], but whether they are appropriate to the paternal perinatal period remains unclear and requires further validation.

### 4.4. Looking Beyond Internal Reliability

Measures of internal reliability were the most frequently reported evidence in the reviewed studies of the psychometric properties of each of the included instruments. The most frequent indication of internal reliability was the Cronbach’s alpha statistic. While reporting internal reliability is important, it alone cannot justify whether the selected instrument is appropriate or superior to other measures. High levels of internal reliability, therefore, indicate that responses to items on an instrument are correlated but cannot be used to infer what underlying construct is being assessed—a fundamental component of an effective instrument. Additionally, measures of internal reliability are unable to be used to make inferences about the temporal stability of the measure (e.g., an instrument’s test re-test reliability), nor how consistent different reporters (e.g., self-report vs. partner-report vs. clinician report) are in their reporting (e.g., inter-rater reliability). These facets might be equally relevant when appraising the quality of an instrument.

Internal reliability can be calculated using data collected during administration, with no further effort on the side of the cohort necessary. Comparatively, test re-test reliability requires re-administration at a later point (e.g., a longitudinal design), whilst inter-rater reliability would require another person (e.g., a partner or a clinician) to complete an assessment of the person’s functioning using a different version of the same tool. It is, therefore, perhaps unsurprising that this thorough assessment of instrument reliability is typically reserved when measures are first developed. Though it will require some additional resourcing to observe, greater attention to these other dimensions of reliability for both new and existing measures used in paternal populations is recommended, as the extent to which previous assessments of reliability derived from different populations are upheld in the paternal population is not well-established.

The assessment of instrument validity is typically reserved for validation studies and less frequently reported in other research (e.g., epidemiological, experimental, or field studies). This is because most forms of validity testing require additional investment from the population of interest, such as completing additional scales or instruments (e.g., to assess criterion validity) or other methodologies—like qualitative interviews or consumer reference groups. One exception is the use of factor analysis methodologies to evaluate the construct validity of an instrument, which can be performed using only information derived from the completion of that instrument. This process can reveal the underlying factor structure of an instrument and provide evidence for distinct subscales or one-dimensionality. This process can also aid in identifying how instruments could be modified to suit the needs of different populations, such as different genders or cultural contexts. Our review examined that the Fathers Fear of Childbirth Scale has reported different factor structures across different cultural contexts, which can affect how this construct is assessed across populations [54,62].

### 4.5. The Utility of Screening Measures Not Specific to Fathers in the Perinatal Period

Whether non-specific mental health assessments are sensitive enough to detect and differentiate impairments during the perinatal period remains a relevant area of investigation for clinicians and scholars alike. Moreover, it remains unclear whether instruments that were developed and validated in majority-maternal contexts are sufficient to accurately identify fathers at risk for a perinatal mental health condition. For example, Fisher et al. recently described that the “screening of paternal depression with traditional measures may present an inaccurate depiction of paternal mental health” [22] (p. 842). In response, some researchers have sought to make modifications to the scoring procedures of these well-validated—but maternally minded—instruments, such as the widely practiced modification to the scoring of the EPDS when used in the paternal population [67,71]. However, other researchers have queried whether these modified scoring procedures are sufficiently able to capture fathers at risk for perinatal mental health conditions or whether purpose-built instruments that are grounded in the paternal experience of perinatal mental health are necessary [86].

The opportunity to administer multiple screening instruments for mental health is unlikely in busy clinical environments where these screening instruments would be deployed. This practice might introduce new dilemmas, such as what to do when scores are divergent on different measures. To exemplify, research by Madsen and Juhl involving a community sample of new fathers found that 5% scored in the clinical range on the EPDS [86], with 3.4% of the sample scoring in the clinical range on the Gotland Male Depression Scale. However, only 2.1% of the sample scored in the clinical range in both assessments. Building upon this work was a more recent study by Psouni et al. [71], who administered the EPDS, Gotland Male Depression Scale, and Beck Depression Inventory-II in a sample of 447 Swedish fathers of children aged 18 months or younger. The study authors similarly identified different proportions of their cohort scoring above the cut-off scores for the EPDS (35.8%), Gotland Male Depression Scale (21.21%), and Beck Depression Inventory-II (27.9%). Taken together, these findings suggest that standard screening instruments of mental health may not always align in the identification of at-risk fathers and, by extension, impact their ability to be appropriately supported. A noteworthy approach the authors took was to statistically integrate items from the EPDS and Gotland Male Depression Scale, resulting in the creation of the Edinburgh-Gotland Depression Scale (EGDS). The result was a hybrid scale that reported superior sensitivity and comparable specificity to either measure in isolation. This method provided a novel attempt to address prior recommendations that postnatal and perinatal mental health screening in men constitutes some items congruent with the ‘male experience’ of depression [86]. However, whether this finding can be replicated in additional cohorts is subject to further research.

The development of instruments specific to fathers in the perinatal period appears to be an increasingly popular area of research. In a prior meta-review of instruments assessing paternal perinatal mental illness, just one was identified to have been designed specifically for fathers in the perinatal period—Paternal Adjustment and Paternal Attitudes Questionnaire [37,90]. This scoping review identified four additional instruments, including the Fathers Postnatal Health Instrument Questionnaire [78], Father’s Fear of Childbirth Scale [87,88], First Time Fathers Experience of Childbirth [75], and Perinatal Assessment of Paternal Affectivity (PAPA) [21]. More recently, measures of pregnancy-related anxiety [115] and perceiving a traumatic childbirth [116] have been developed specifically for fathers. The development of these instruments represents important progress in responding appropriately to fathers during the perinatal period.

### 4.6. Limitations

While this study is comprehensive, it does have some limitations. First, restricting peer-reviewed articles published in English-language journals resulted in a focus on research from industrialized Western populations, which may reflect traditional Western norms and customs regarding fatherhood, masculinity, and mental health. Thus, the relevance of these findings to different cultural contexts, including non-Western populations, remains unclear and is a subject for further inquiry. Second, while a rigorous methodology was followed, our scoping review methodology still has some shortcomings. For example, we searched for studies using four popular databases. Recent work has shown that Embase tends to produce the most unique references, with the number of relevant studies shown to be enhanced by the use of Medline [117]. Our inclusion of PsycInfo and Proquest also helped to find discipline-specific studies. However, it is possible that the inclusion of additional databases (e.g., Web of Science) or search methods (e.g., gray literature) may identify other relevant scales. Additionally, we followed earlier guidelines for scoping reviews [118] that described that whilst reasons for the exclusion of records at the full-text screening are required, reasons at the title and abstract screening phase were not. Accordingly, this was not completed in the current scoping review, but could be carried out in future to adhere to the latest recommendations. Future studies may wish to include additional methods of finding eligible studies. Limitations of scoping review methods include the absence of methodological and risk of bias evaluations, which reduce the thoroughness and scope of the recommendations that can be made based on the results. Additionally, the literature search was conducted in July 2023 and it is possible that recent key publications have not been included in the discussion of the results. Whilst a delay in the time between initial search to final publication is expected in evidence synthesis research, it is nevertheless important to consider the potential emergence of new measures of fathers’ perinatal mental health and wellbeing during this period. However, recent developments within the field have often involved the validation of existing scales captured in our review in new contexts. Rather than the development of new scales entirely. For example, Cortés and colleagues [110] recently described a validation of the PAPA [21] among Chilean fathers.

## 5. Conclusions

Growing awareness of the mental health needs of fathers has driven efforts towards the timely and accurate identification of at-risk individuals to provide better opportunities for intervention and prevention efforts. Accurate identification of paternal mental health difficulties across the perinatal period can only be assured with access to instruments that are well-validated for use in the paternal population during this time. This scoping review helped to fill a gap by mapping currently available tools used to assess paternal perinatal mental health and their psychometric properties. Several purpose-built instruments, such as the PAPA, were also identified. These purpose-built measures produced promising results as screening instruments for psychosocial distress (beyond just depressive symptoms). However, further evaluation is necessary.

## Figures and Tables

**Table 1:** Included studies (*n* = 36).

Year of Publication	Title	Authors	Reference
2023	The latent factor structure and assessment of childbirth-related PTSD in fathers: Psychometric characteristics of the City Birth Trauma Scale—French version (partner version)	Sandoz et al.	[53]
2023	Psychometric properties of the Chinese version of the fathers’ fear of childbirth scale: A cross-sectional study.	Guo et al.	[54]
2022	A systematic review and meta-analysis of studies validating Edinburgh Postnatal Depression Scale in fathers	Shafian et al.	[35]
2022	Validation of the Karitane Parenting Confidence Scale in measuring parental self-efficacy of Australian fathers	Wright et al.	[55]
2022	The Perinatal Assessment of Paternal Affectivity (PAPA): Italian validation of a new tool for the screening of perinatal depression and affective disorders in fathers	Baldoni et al.	[21]
2022	Digital screening for postnatal depression: Mixed methods proof-of-concept study	Eisner et al.	[56]
2022	Validity and Reliability of the Chinese Version of the Edinburgh Postnatal Depression Scale for Fathers of Newborns	Jen et al.	[57]
2021	Screening for early signs of paternal perinatal affective disorder in expectant fathers: A cluster analysis approach	Mangialavori et al.	[58]
2021	Development and validation of a measure of birth-related PTSD for fathers and birth partners: The City Birth Trauma Scale (Partner Version)	Webb et al.	[59]
2021	Depressive symptoms in men immediately after birth.	Abdollahi et al.	[60]
2021	Psychometric properties of the Pregnancy-Related Anxiety Scale for use with fathers during pregnancy	Cameron et al.	[61]
2021	Design and psychometric evaluation of the fathers’ fear of childbirth scale: A mixed method study	Ghaffari et al.	[62]
2020	Screening for mood difficulties in men in Italy and Australia using the Edinburgh Postnatal Depression Scale and the Matthey Generic Mood Questionnaire	Matthey and Vedova	[63]
2019	Self- screening using the Edinburgh post natal depression scale for mothers and fathers to initiate early help seeking behaviours	Edward et al.	[64]
2018	Prevalence and determinants of antepartum depressive and anxiety symptoms in expectant mothers and fathers: Results from a perinatal psychiatric morbidity cohort study in the east and west coasts of Malaysia	Nasreen et al.	[65]
2018	The Birth Experiences Questionnaire: A brief measure assessing psychosocial dimensions of childbirth	Saxbe et al.	[66]
2018	Paternal Perinatal Depression Assessed by the Edinburgh Postnatal Depression Scale and the Gotland Male Depression Scale: Prevalence and Possible Risk Factors	Carlberg et al.	[67]
2018	Assessment of postpartum depression in a group of Chilean Parents.	Francisca Pérez et al.	[68]
2018	Universal Postpartum Mental Health Screening for Parents of Newborns With Prenatally Diagnosed Birth Defects.	Cole et al.	[69]
2017	Paternal Adjustment and Paternal Attitudes Questionnaire: Antenatal and postnatal Portuguese versions	Pinto et al.	[70]
2017	Symptoms of depression in Swedish fathers in the postnatal period and development of a screening tool	Psouni et al.	[71]
2015	Assessing birth experience in fathers as an important aspect of clinical obstetrics: How applicable is Salmon’s Item List for men?	Gawlik et al.	[72]
2015	The Edinburgh postnatal depression scale for fathers: A contribution to the validation for an Italian sample	Loscalzo et al.	[73]
2013	How well does the Edinburgh Postnatal Depression Scale identify depression and anxiety in fathers? A validation study in a population based Swedish sample	Massoudi et al.	[74]
2012	Father for the first time—Development and validation of a questionnaire to assess fathers’ experiences of first childbirth (FTFQ)	Premberg et al.	[75]
2012	Validation of three psychometric instruments for screening for perinatal common mental disorders in men in the north of Vietnam	Tran et al.	[76]
2012	Partner report of paternal depression using the Edinburgh Postnatal Depression Scale-Partner.	Fisher et al.	[77]
2011	The development of two postnatal health instruments: One for mothers (M-PHI) and one for fathers (F-PHI) to measure health during the first year of parenting	Jones et al.	[78]
2010	Depression in fathers in the postnatal period: Assessment of the Edinburgh Postnatal Depression Scale as a screening measure	Edmondson et al.	[79]
2010	Detecting postnatal depression in Chinese men: A comparison of three instruments.	Lai et al.	[80]
2009	The use of Edinburgh Postnatal Depression Scale to identify postnatal depression symptoms at well child visit.	Currò et al.	[81]
2008	Using the Edinburgh Postnatal Depression Scale to screen for anxiety disorders	Matthey	[82]
2008	Comparisons of different instruments to measure blues and to predict depressive symptoms 2 months postpartum: A study of new mothers and fathers	Edhborg	[83]
2008	Psychosocial assessment of expectant fathers	Fletcher et al.	[84]
2007	Parents’ postnatal sense of security (PPSS): Development of the PPSS instrument	Persson et al.	[85]
2007	Paternal depression in the postnatal period assessed with traditional and male depression scales	Madsen and Juhl	[86]

**Table 2:** Summary characteristics of included studies (*n* = 36).

Year of Publication, Country, Number of Study Participants, Recruitment Context, and Time of Measurement	*n* (% of Included Studies)
Year of publication	
2003–2008	5 (13.89)
2009–2014	8 (22.22)
2015–2020	11 (30.56)
2021–2023	12 (33.33)
Country	
Australia	4 (11.11)
Canada	1 (2.78)
Chile	1 (2.78)
China	2 (5.56)
Denmark	1 (2.78)
Germany	1 (2.78)
Iran	2 (5.56)
Italy	4 (11.11)
Malaysia	1 (2.78)
Portugal	1 (2.78)
Sweden	6 (16.67)
Taiwan	1 (2.78)
United Kingdom	4 (11.11)
United States	3 (8.33)
Vietnam	1 (2.78)
Multiple countries	3 (8.33)
Number of study participants	
<50	2 (5.56)
50–99	4 (11.11)
100–499	23 (63.89)
500–1000	5 (13.89)
>1000	2 (5.56)
Recruitment context	
Hospital clinics	16 (44.44)
Primary/community care	10 (27.78)
Other (e.g., flyers, online surveys, registries, etc.)	10 (27.78)
Time of measurement ^a^	
Pregnancy	9 (25.00)
0–6 months postpartum	18 (50.00)
6–12 months postpartum	9 (25.00)

^a^ Studies that assessed perinatal mental health across multiple period, were assigned to the upper age-bracket.

**Table 3:** Instruments measuring perinatal mental health (*n* = 14).

Instrument (Year of Publication)	Form/Version	Mental Health Domains Assessed	Number of Items	Timeframe	Response Options (Score Range)	Developed For/Aim/Context	Informant	Example Item	Described in
Birth Experiences Questionnaire (2018) [66]	BEQ	Stress, fear, partner support during birth	10	1–2 days post-partum	1 (not at all) to 7 (extremely)	To assess stress, fear, and partner support during birth.	Self-report	Did you fear for your partner’s life?	[66]
Edinburgh Postnatal Depression Scale (1987) [31]	EPDS (multiple languages) (digital and print versions)	Postpartum Depression (PPD)	10	Past seven days	Variety of response options	Screen for emotional distress during pregnancy and postpartum period	Self-report	I have been anxious or worried for no good reason	[35,53,56,57,60,63,64,65,67,68,71,73,74,76,79,80,81,82,83]
							Maternal report of partner		[76]
Fathers Postnatal Health Instrument Questionnaire (2011) [78]	F-PHI	Postnatal health	27	First twelve months post-partum	0 (never) to 4 (always)	To assess the positive and negative aspects of health in the first twelve months post-partum.	Self-report	My baby makes me feel full of great joy	[78]
Father’s Fear of Childbirth Scale (1998) [87,88]	FFCS	Tokophobia	17	-	1 (I don’t agree at all) to 5 (I completely agree)	To investigate the fear of childbirth amongst fathers.	Self-report	I’m afraid that my spouse’s childbirth will be risky.	[62]
	C-FFCS (Chinese version)								[54]
First Time Fathers Experience of Childbirth (2012) [75]	FTFQ	Worry, emotional support, acceptance	22	-	1 (strongly agree) to 4 (disagree)	Assessment of the experiences of first-time fathers.	Self-report	I felt accepted at the delivery ward	[75]
Karitane Parenting Confidence Scale (2008) [89]	KPCS	Parent Self-Efficacy	15	-	0 (no, hardly ever) to 3 (yes, most of the time)	Assessment of parenting efficacy in parents of young children aged 0–12 months.	Self-report	I am confident about feeding my baby	[55]
Parents Postnatal Sense of Security Instrument (2007) [85]	PPSS	Sense of security	13	First post-natal week	1 (strongly disagree) to 4 (strongly agree)	Assess post-natal sense of security within the first week of childbirth	Self-Report	I felt that I participated in general	[85]
Paternal Adjustment and Paternal Attitudes Questionnaire (1992) [90]	Antenatal (PAPA-AN) and Postnatal Version (PAPA-PN)	Paternal adjustment and attitudes during transition to parenthood	30	-	1 (never) to 4 (very often).	Assess paternal adjustment and paternal attitudes during the transition to parenthood	Self-report	Have you been worrying that you might not be a good father?	[70]
Perinatal Assessment of Paternal Affectivity (2022) [21]	PAPA	Paternal Depressive and Affective Disorder	8 + 2 open questions	Two weeks	0 (not at all) to 3 (a lot)	Identify fathers at risk of developing a perinatal affective disorder	Self-report	I have had some problems with sleeping, eating or sexual desire	[21]
Pregnancy-Related Anxiety Scale (2019) [91]	PRAS	Pregnancy Anxiety	10	-	1 (never or not at all) to 4 (a lot of the time or very much)	The extent to which fathers worry about pregnancy related concerns	Self-report	I am concerned or worried about losing the baby.	[61]
Psychosocial Questionnaire (2008) [84]	IPC Questions	Psychosocial Difficulties	14	Up to 12 months	Variety of responses, e.g., yes/no	Identifying fathers needs	Self-report	I will be able to provide financial support for my family	[84]
Salmon’s Item List (1990) [92,93]	German Version (SIL-Ger)	Birth Experiences	20	4–6 weeks postpartum	Binary selection option	Birth experiences of fathers	Self-report	Disappointed or not disappointed	[72]
The Blues Questionnaire (1989) [94]	Blues Questionnaire	Blues Symptoms within first week postpartum	28	First week postpartum	Yes/no	Incidence of postpartum blues	Self-report	Mentally tense (yes or no)	[83]
The City Birth Trauma Scale (2018) [95]	City BiTS (Partner Version) City BiTS French Partner Version	Birth-related PTSD	29	Symptoms in past week up to six months post-partum	Variety of responses zero (not at all) to three (five or more times)	To assess DSM-V criteria of PTSD in birth partners	Self-report	Getting upset when reminded of the birth	[53,59]

**Table 4:** Instruments measuring general mental health (*n* = 14).

Instrument (Year of Publication)	Form/Version	Mental Health Domains Assessed	Number of Items	Timeframe	Response Options (Score Range)	Developed For/Aim/Context	Informant	Example Item	Described in
Beck Depression Inventory (1961) [96]	BDI-I (multiple languages)	Depression	21	Past week	0–3 (various response options)	To measure symptoms of depression in clinical and non-clinical samples	Self-report	I have lost confidence in myself	[57,68,80]
	BDI-II								[57,71,73]
Center for Epidemiologic Studies Depression Scale (1977) [97]	CES-D	Depression	20	Past week	1 (rarely or never) to 4 (always or almost always)	To assess symptoms of depression	Self-report	I thought my life had been a failure.	[58,69,73]
General Health Questionnaire (1988) [98]	GHQ-12	Mental illness	12	Past few weeks	Bimodal and 4-point Likert scale	Severity of mental health problems	Self-report	Have you recently lost much sleep over worry?	[76]
Gotland Male Depression Scale (2002) [99]	GMDS	Depression (in Males)	13	-	0 (not present) to 3 (present to a high degree)	Detect major depression in men	Self-report	I/others have noticed that I am more aggressive, outward reacting, difficulties keeping self-control	[67,71,86]
Hospital Anxiety and Depression Scale (1983) [100]	HADS-A (Anxiety Subscale only) French Version	Anxiety symptoms	7	Past week	4-point Likert scale (varying responses)	Screen for anxiety	Self-report	I get sudden feelings of panic	[53]
	HADS-A (Swedish Version)								[74]
Impact of Event Scale-Revised (2007) [101]	IES-R	Psychological and behavioral distress	22	Past seven days	0 (not at all) to 4 (extremely)	Measure of post-traumatic stress disorder symptoms	Self-report	Pictures about it popped into my mind	[69]
Kessler Psychological Distress Scale (1992) [102]	K-10	Psychological Distress	10	Past four weeks	1 (none of the time) to 5 (all of the time)	To screen for non-specific psychological distress	Self-report	About how often did you feel that everything was an effort?	[64]
Matthey Generic Mood Questionnaire (2013) [103]	MGMQ	Distress	4	Past two weeks	Various response formats	Screen for mood	Self-Report	Have you felt very stressed, anxious, or unhappy, or found it difficult to cope, for some of the time?	[63]
Patient Health Questionnaire: Depression Module (2001) [104]	PHQ-9 (Chinese Version)	Depression	9	Past two weeks	0 (not at all) to 4 (nearly every day)	Screens for severity of depression	Self-report	Feeling tired or having little energy	[80]
Perceived Stress Scale (2001) [104]	PSS	Stress	10	Last six months	0 (never) to 4 (very often)	Perception of stress	Self-Report	In the last month, how often have you felt difficulties were piling up so high that you could not overcome them?	[58]
Post Traumatic Stress Disorder Checklist for DSM-V (2015) [105]	PCL-5	Posttraumatic Stress Disorder	20	Past month	0 (not at all) to 4 (Extremely)	Screens for PTSD	Self-report	Repeated, disturbing, and unwanted memories of the stressful experience?	[53]
The Symptom Checklist 90-Revised (1983) [106]	SCL-90-R	Psychiatric Symptomology	90	Past week	0 (not at all) to 4 (extremely)	Assess a broad range of psychological problems	Self-Report	Feeling lonely even when you are with people	[58]
Visual Analogue Scales (1921) [107]	VAS	Depressed mood, anxiety, tiredness, happiness	4	Past five days	100 mm line	Assess severity of symptoms	Self-report indicate response on a continuum	Tiredness	[83]
Zung’s Self-Rated Anxiety Scale (1971) [108]	Zung SAS	Anxiety and depression	20	-	1 (a little of the time) to 4 (most of the time)	Detection of anxiety disorder	Self-report	I can feel my heart beating fast.	[76]

**Table 5:** Reliability and validity of perinatal mental health instruments (*n* = 14).

Instrument	Internal Reliability	Test Re-Test Reliability	Inter-Rater Reliability	Construct Validity	Criterion Validity	Other Evaluations of Psychometric Properties	Source/s of Evidence
Birth Experiences Questionnaire	α = 0.80	-	-	Unifactorial structure supported by exploratory factor analysis.	Father BEQ not significantly correlated with: Father pregnancy anxiety scale Father prenatal stress Father prenatal depression Father prenatal social support Father BEQ correlated with maternal BEQ total	-	[66]
Edinburgh Postnatal Depression Scale	α = 0.65–0.91	-	-	Exploratory and confirmatory analysis support structure.	Correlated with: F-PHI PAPA-AN PRAS The Blues Questionnaire BDI-II GMDS CES-D VAS HADS-A (French) PCL-5 City BiTs (French)	Split half Spearman–Brown coefficient was 0.84. Specificity and sensitivity cut-offs were established. App and paper version both have shown perfect agreement for EPDS thresholds	[53,57,60,65,67,68,71,73,74,76,77,80,82]
Fathers Postnatal Health Instrument Questionnaire	α = 0.72 (relationship with baby) α = 0.72 (relationship with partner) α = 0.76 (support from partner) α = 0.90 (support from friends) α = 0.89 (Mood) α = 0.82 (Role as partner)	T1-T2 = One Week *r* = 0.60 (relationship with baby) *r* = 0.74 (relationship with partner) *r* = 0.76 (support from partner) *r* = 0.60 (support from friends) *r* = 0.87 (Mood) *r* = 0.88 (Role as partner)	-	Items first derived from qualitative interviews. Multidimensional structure supported by exploratory factor analysis.	Correlated with: EPDS WEMWBS MCS PCS	-	[78]
Father’s Fear of Childbirth Scale	α = 0.84 to 0.92/ω = 0.93 (Overall) α = 0.86 to 0.91/ω = 0.91 to 0.93 (Factor 1) α = 0.87 to 0.86/ω = 0.87 to 0.88 (Factor 2) α = 0.88/ω = 0.88 (Factor 3)	-	-	Qualitative consultation with medical experts and community. Factor structure has been tested using both exploratory and confirmatory factor analytic approaches.	Correlated with: CAQ FOBS	Face validity assessed via consumer engagement and feedback. Convergent and discriminant validity assessed. Cross-cultural validity was also assessed via multigroup confirmatory factor analysis.	[54,62]
First Time Fathers Experience of Childbirth	α = 0.82 (worry) α = 0.73 (information) α = 0.65 (emotional support) α = 0.66 (Acceptance)	-	-	Known groups validation used to assess discriminant validity.	-	Face validity assessed via pilot in fathers.	[75]
Karitane Parenting Confidence Scale	α = 0.80 (parenting tasks) α = 0.66 (parenting role) α = 0.80 (total scale)	-	-	Confirmatory factor analysis used.	-	-	[55]
Parents Postnatal Sense of Security Instrument	α = 0.89 (empowering behavior) α = 0.68 (mother’s wellbeing) α = 0.76 (general wellbeing) α = 0.62 (affinity in the family) α = 0.77 (total scale)	-	-	Exploratory factor analysis used.	Correlated with a specific question about experienced security in the first week post-partum	Face validity assessed by midwives	[85]
Paternal Adjustment and Paternal Attitudes Questionnaire	PAPA-AN α = 0.82 (attitudes towards sex) α = 0.74 (marital relationship) α = 0.71 (attitudes towards pregnancy and baby) α = 0.91 (total scale) PAPA-PN α = 0.81 (attitudes towards sex) α = 0.87 (marital relationship) α = 0.74 (attitudes towards pregnancy and baby) α = 0.90 (total scale)	-		Longitudinal confirmatory factor analysis used.	Correlated with: State-Trait Anxiety Inventory EPDS	Clinical validity assessed and suggested excellent accuracy power	[70]
Perinatal Assessment of Paternal Affectivity	ω = 0.86/ordinal α = 0.85	Three-month interval ICC = 0.59 (95% CI 0.41–0.72)	-	Confirmatory factor analysis	Correlated with CES–D SCL-90-R PSS Dyadic Adjustment Scale		[21]
Pregnancy-Related Anxiety Scale.	α = 0.87		-	Exploratory factor analysis	Correlated with: STAI EPDS	Predictive validity assessed PRAS significantly predicted EPDS one-month postpartum	[61]
Psychosocial Questionnaire	-	-	-	-	-	-	[84]
Salmon’s Item List	α = 0.87	-	-	Exploratory factor analysis	-	-	[72]
The Blues Questionnaire	-	-	-	-	Correlated with: EPDS VAS	Predictive utility compared to EPDS two months later	[83]
The City Birth Trauma Scale	α = 0.78 (stressor) α = 0.87 (re-experiencing) α = 0.82 (avoidance) α = 0.86 (negative cognitions and mood) α = 0.87 (hyperarousal) α = 0.89–0.94 (total scale)	-	-	Exploratory and confirmatory factor analysis	Correlated with: PCL-5 HADS-A (French) EPDS (French)	Readability of the scale assessed with Flesch readability scale and Gunning Fog index Known-group validity in fathers who met PTSD criteria vs. those that did not	[53,59]

**Table 6:** Reliability and validity of general mental health instruments (*n* = 14).

Instrument	Internal reliability	Test Re-Test Reliability	Inter-Rater Reliability	Construct Validity	Criterion Validity	Other	Source/s of Evidence
Beck Depression Inventory	α = 0.86–0.95	-	-	Receiver operator characteristic calculated as satisfactory.	Correlated with: EPDS	Split half reliability measured by Spearman–Brown coefficient was 0.85	[57,68,71,73,80]
Center for Epidemiologic Studies Depression Scale	α = 0.71–0.86	-	-		Correlated with: EPDS	-	[58,69,73]
General Health Questionnaire	α = 0.70	-	-	Receiver operator characteristic calculated as satisfactory.	-	-	[76]
Gotland Male Depression Scal	α = 0.84–0.88	-	-		Correlated with: EPDS	Scale was combined with the EPDS to assess depressive symptoms in fathers. EFA used.	[67,71,86]
Hospital Anxiety and Depression Scale (Anxiety subscale only)	α = 0.78	-	-	-	Correlated with: EPDS (French) PCL-5 City BiTs (French)	-	[53]
Impact of Event Scale-Revised	α = 0.93	-	-	-	-	-	[69]
Kessler Psychological Distress Scale	-	-	-	-	-	-	[64]
Matthey Generic Mood Questionnaire	-	-	-	-	-	-	[63]
Patient Health Questionnaire: Depression Module	α = 0.88 *	-	-	Receiver operator characteristic calculated as satisfactory.	-	Split half reliability measured by Spearman–Brown coefficient was 0.82 Area under curve 0.92	[80]
Perceived Stress Scale	α = 0.76 *	-	-	-	Correlated with: CES-D SCL-90-R subscales The Dyadic Adjustment Scale	-	[58]
Post Traumatic Stress Disorder Checklist for DSM-V	α = 0.89 *	-	-	-	Correlated with: City BiTs (French) HADS-A EPDS		[53]
The Symptom Checklist 90-Revised	α = 0.72 (anxiety) α = 0.78 (somatization) α = 75 (hostility)	-	-	-	All subscales correlated with: CES-D49 PSS The Dyadic Adjustment Scale	-	[58]
Visual Analogue Scales	-	-	-	-	Correlated with: EPDS Blues Questionnaire	-	[83]
Zung’s Self-Rated Anxiety Scale	α = 0.80	-	-	Receiver operator characteristic calculated as satisfactory.	-	-	[76]

* Statistic is derived from a combined maternal and paternal group.

## Data Availability

The original contributions presented in this study are included in the article/Supplementary Materials. Further inquiries can be directed to the corresponding author.

## Supplementary Materials

The following supporting information can be downloaded at: https://www.mdpi.com/article/10.3390/ijerph22071126/s1. Supplementary File: GPT-3.

## Abbreviations

The following abbreviations are used in this manuscript:

COPE	Australia’s Centre of Perinatal Excellence
EGDS	Edinburgh-Gotland Depression Scale
EPDS	Edinburgh Postnatal Depression Scale
GPT	generative predictive text
JBI	Joanna Briggs Institute
OSF	Open Science Framework
PAPA	Perinatal Assessment of Paternal Affectivity
PND	Postnatal depression
PPND	Paternal postnatal depression
