# Supplementary material for: Screening Measures of Perinatal Mental Health and Wellbeing in Fathers: A Scoping Review
Source: Int J Environ Res Public Health. 2025 Jul 16;22(7):1126. doi: 10.3390/ijerph22071126 (PMC12294220; doi:10.3390/ijerph22071126)
Supplement: Supplementary file 1 [file ijerph-22-01126-s001.zip › ijerph-3681468-supplementary.pdf]

**GPT-3**

**Input**

I am conducting a scoping review. The review will be about assessment tools used to measure perinatal mental health and adjustment used in samples of men, males, fathers, and dads. Generate search terms for Embase, Medline, ProQuest, and PsycINFO

**Output**

Suggested Search Terms:

1. Embase (via Ovid)

('perinatal period'/exp OR perinatal* OR prenatal* OR antenatal* OR postnatal* OR postpartum*) AND ('mental health'/exp OR 'mental health' OR 'depression'/exp OR depression OR 'anxiety'/exp OR anxiety OR 'psychological distress' OR adjustment) AND ('father'/exp OR father* OR dad* OR male* OR men) AND ('assessment'/exp OR assessment OR screening OR measurement OR questionnaire OR scale OR tool OR instrument)

2. Medline (via PubMed)

("Perinatal Care"[Mesh] OR perinatal* OR prenatal* OR antenatal* OR postnatal* OR postpartum*) AND ("Mental Health"[Mesh] OR "Depression"[Mesh] OR "Anxiety"[Mesh] OR mental health OR depression OR anxiety OR psychological distress OR adjustment) AND ("Fathers"[Mesh] OR father* OR dad* OR male* OR men) AND ("Psychological Tests"[Mesh] OR assessment OR screening OR measurement OR questionnaire OR scale OR tool OR instrument)

3. ProQuest (e.g., APA PsycInfo, Health & Medical Collection)

TI,AB(perinatal* OR prenatal* OR antenatal* OR postnatal* OR postpartum*) AND TI,AB(mental health OR depression OR anxiety OR psychological distress OR adjustment) AND TI,AB(father* OR dad* OR male* OR men) AND TI,AB(assessment OR screening OR measurement OR questionnaire OR scale OR tool OR instrument)

4. PsycINFO (via Ovid)

(perinatal* OR prenatal* OR antenatal* OR postnatal* OR postpartum*) AND (mental health OR depression OR anxiety OR psychological distress OR adjustment) AND (father* OR dad* OR male* OR men) AND (assessment OR screening OR measurement OR questionnaire OR scale OR tool OR instrument)
